# Should the PBL tutor be present? A cross-sectional study of group effectiveness in synchronous and asynchronous settings

**DOI:** 10.1186/s12909-020-02018-3

**Published:** 2020-03-31

**Authors:** Samuel Edelbring, Siw Alehagen, Evalotte Mörelius, AnnaKarin Johansson, Patrik Rytterström

**Affiliations:** 1 School of Health Sciences, Örebro University, 701 82 Örebro, Sweden; 2 Department of Health, Medicine and Caring Sciences (HMV), Linköping University, 581 83 Linköping, Sweden; 3 School of Nursing and Midwifery, Edith Cowan University, 270 Joondalup Drive, Joondalup, Western Australia Australia

**Keywords:** Problem-based learning, Tutorial groups, Tutoring

## Abstract

**Background:**

The tutorial group and its dynamics are a cornerstone of problem-based learning (PBL). The tutor’s support varies according to the setting, and it is pertinent to explore group effectiveness in relation to different settings, for example online or campus-based. The PBL groups’ effectiveness can partly be assessed in terms of cognitive and motivational aspects, using a self-report tool to measure PBL group effectiveness, the Tutorial Group Effectiveness Instrument (TGEI).

This study’s aim was to explore tutor participation in variations of online and campus-based tutorial groups in relation to group effectiveness. A secondary aim was to validate a tool for assessing tutorial group effectiveness in a Swedish context.

**Methods:**

A cross-sectional study was conducted with advanced-level nursing students studying to become specialised nurses or midwives at a Swedish university. The TGEI was used to measure motivational and cognitive aspects in addition to overall group effectiveness. The instrument’s items were translated into Swedish and refined with an expert group and students. The responses were calculated descriptively and compared between groups using the Mann–Whitney U and Kruskal–Wallis tests. A psychometric evaluation was performed using the Mokken scale analysis. The subscale scores were compared between three different tutor settings: the tutor present face-to-face in the room, the tutor present online and the consultant tutor not present in the room and giving support asynchronously.

**Results:**

All the invited students (*n* = 221) participated in the study. There were no differences in motivational or cognitive aspects between students with or without prior PBL experience, nor between men and women. Higher scores were identified on cognitive aspects (22.6, 24.6 and 21.3; *p* < 0.001), motivational aspects (26.3, 27 and 24.5; *p* = 002) and group effectiveness (4.1, 4.3, 3.8, *p* = 0.02) for the two synchronously tutored groups compared to the asynchronously tutored group. The TGEI subscales showed adequate homogeneity.

**Conclusions:**

The tutor’s presence is productive for PBL group effectiveness. However, the tutor need not be in the actual room but can provide support in online settings as long as the tutoring is synchronous.

## Background

The tutorial group and its dynamics are fundamental in the problem-based learning (PBL) educational approach. This dynamic is influenced by the tutor and how he or she interacts with the group [1]. The tutor’s roles range from influencing the learning climate to evaluating parts of the learning process [2]. At a time when higher education faces economic constraints and flexible digital opportunities are suggested for PBL, the tutor’s characteristics and contributions need informed consideration. Online resources have opened up many possibilities, and distance and blended settings are increasingly being used in health professions’ education, with satisfying results both in individual and collaborative settings of inquiry-based learning [3–5]. The main appeal is their flexibility regarding time and place. However, the online setting changes the context for both small group dynamics and tutor interactions [6, 7].

Collaborative work in the small group setting has formed part of the PBL approach since its early beginnings [8]. In its traditional format, the tutorial group consists of a maximum of eight students and a tutor, who meet in a designated room at a scheduled time [8, 9]. The small group has been considered to benefit the student learning process in motivational, social cohesion, cognitive and developmental perspectives [10, 11]. It is reasonable to assume that variations to the classic tutorial setting have implications for the tutorial group’s cognitive and motivational aspects. As a consequence of using digital technology, the traditional PBL arrangements have been re-structured and thereby need a renewed awareness of the tutor and the setting’s influence on group dynamics.

Several PBL aspects have been influenced by digital technology [12–14]. For example, instead of library visits, internet access provides immediate access to information, and the replacement of paper cases with virtual patients has influenced study practices [15]. In general, technology adds value to the PBL process, such as accessibility, and it can be a way of revitalising the PBL learning situation [5, 16–18]. The learning process, and not only its direct outcomes, is of interest in the PBL approach. Thus, it is of value to study the tutorial group’s perceptions of cognitive and motivational aspects in different settings.

Slavin [10] highlights two major complementary perspectives: the motivational perspective, which focuses on the students’ responsibility for one another’s achievements and reward and goal structures and the cognitive perspective, which involves the group’s mental processing of information. These two aspects were operationalised into an instrument used to assess PBL tutorial groups based on their own ratings, the Tutorial Group Effectiveness Instrument (TGEI) [19]. Educators and researchers can use the TGEI to gain knowledge about group effectiveness in different PBL settings [20]. Few studies have reported these aspects by comparing face-to-face groups with synchronous and asynchronous online tutorial groups.

The aim of this study was to explore the role of tutor participation in variations of online and campus-based tutorial groups in relation to group effectiveness. A secondary aim was to validate a tool for assessing tutorial group effectiveness in a Swedish context.

## Methods

### Setting

The study was performed at a university medical faculty in southeast Sweden. PBL has been the pedagogical philosophy in all programmes and courses at this faculty since 1987. At the time of the study, there were eight specialist nursing programmes: midwifery, anaesthesia, intensive, surgical, medical, paediatric, public health and psychiatric care. The programmes are all at advanced level and comprise 60–90 credits, i.e. two to three semesters of full-time studies. Half of the programmes are offered as part-time studies.

Collaboration with other academic institutions has enabled study groups in other geographical areas. These students can carry out their clinical training in hospitals and health care clinics in their home counties as well as part of their theoretical studies. However, some parts of the education are only offered at the university campus.

Participation in tutorial groups was possible in three different ways:

A. Traditionally, with scheduled meetings when all the students and the tutor meet in the same room.
B. The students and the tutor participate in synchronous sessions from different locations via the internet.
C. The students meet in the same room, and the tutor works as a consultant and gives the students comments asynchronously and digitally.

### Participants

The participants were students, all registered nurses, attending one of the eight specialist nursing programmes. They were attending end of their first, second (fulltime students), or fourth (for 50% part-time students) semester. Thus, the questionnaires were answered in the middle (40% of the participants) and the end (60% of the participants) of the programme.

### Instrument

The TGEI was used, as it has been shown to be valid and reliable [19]. The instrument includes three subscales that measure different aspects of tutorial group learning and an overall rating of group effectiveness. The three aspects are cognitive (7 items), motivational (7 items) and demotivational (6 items). The demotivational scores are reversed in the analysis because of negative wording. The items are graded on a 5-point Likert scale, ranging from 1 = strongly disagree to 5 = strongly agree. The last item asks the student to rate the overall effectiveness of the tutorial group from 1 to 5, where 1 is insufficient and 5 is excellent.

The validation of the Swedish version was carried out in four steps:

1. The items were translated into Swedish by a bilingual person working in higher education.
2. The wording was then individually evaluated by three PBL experts and an experienced tutor. Their suggestions were discussed in the research group.
3. The item wording was further discussed in cognitive testing with a representative student group interview.
4. A tutorial group piloted and discussed the latest version before the final version was established. Steps 3 and 4 contributed to the construct validity.

Based on arguments on that back-translation can provide a false security on content validity [21], we chose to rely on content experts and cognitive student testing for content and construct validity.

### Data collection

The TGEI was distributed to the students attending the different programmes in the spring semesters of 2016 and 2017. The paper form was distributed and collected in class; each form was returned in an envelope addressed to the first author, who was not involved in the courses, to ensure anonymity.

The students received written and oral information. They were informed that their participation was voluntary and anonymous and that their answers could not influence their study results. No student abstained from participation.

### Statistics

Descriptive statistics are presented with means, standard deviations (SD), medians, quartiles and percentages. The Mann–Whitney U-test was used for the comparisons between two groups and the Kruskal–Wallis test for the three group comparisons. A *p* value < 0.05 was considered statistically significant.

The psychometric properties regarding reliability and homogeneity were estimated by Cronbach’s alpha and Loevinger’s coefficient *H*. The coefficient *H* estimates homogeneity, i.e. the degree to which a set of items measures the same underlying construct [22]. A coefficient *H* < 0.3 is not considered unidimensional. A coefficient *H* between 0.3 and 0.4 is considered weak, between 0.4 and 0.5 medium and above 0.5 strong. Cronbach’s alpha > 0.7 is considered to reflect adequate internal reliability [23]. IBM SPSS Statistics 25 was used for all tests except for the coefficient *H*, for which R 3.3.2 was used with the Mokken 2.8.10 package.

## Results

A total of 221 students participated in the study (100% response rate). There were 192 (87%) female and 29 (13%) male students, with a mean age of 32.4 (SD 6.2) years. Of the students, 126 (57%) had previous PBL experience, and 94 (43%) had no previous PBL experience. The mean (SD) and median (Q1–Q3) scores for each aspect and for the overall rating of group effectiveness are displayed in Table 1. There was no difference in scores for any of the cognitive or motivational aspects or in the overall rating of group effectiveness between women and men or between students with and without previous PBL experience.

**Table 1:** TGEI subscale scores for all participants

	Items	Mean (SD)	Median (Q1-Q3)
Cognitive aspects	7	22.3 (3.6)	23 (21–24)
Motivational aspects	7	25.9 (4.5)	26 (23–29)
Demotivational aspects	5	10.1 (4.1)	9 (7–13)
Overall rating of group productivity	1	4.0 (0.78)	4 (4–4)

Number of scale items, summed mean, median and corresponding standard deviation and interquartile range

### Comparison of the three tutorial groups

The students participated in one of the following tutorial groups: the tutor was present in the room (A, *n* = 136); the tutor was present online (B, *n* = 17); and the consultant tutor was not present in the room and gave support asynchronously (C, *n* = 68). The summed mean (SD) and median (Q1–Q3) scale scores for each group are shown in Table 2.

**Table 2:** Group comparison of TGEI scores

	(A) Tutor present in the room, *n* = 136		(B) Tutor present Online, *n* = 17		(C) Consultant Tutor, *n* = 68		*p*-value^a^
	Mean (SD)	Median (Q1-Q3)	Mean (SD)	Median (Q1-Q3)	Mean (SD)	Median (Q1-Q3)	
Cognitive aspects	22.6 (3.4)	23 (21–24)	24.6 (2.2)	24 (23.5–25)	21.3 (3.8)	22 (20–23.8)	0.0001
Motivational aspects	26.3 (4.6)	27 (24–30)	27.7 (2.4)	28 (25.5–29.5)	24.5 (4.4)	25 (22–27)	0.002
Demotivational aspects	10.5 (4.2)	10 (7–14)	8.8 (3.7)	8 (6–10.5)	9.6 (3.9)	9 (7–13)	n.s.
Overall rating of group productivity	4.1 (0.79)	4 (4–5)	4.3 (0.7)	4 (4–5)	3.8 (0.7)	4 (3–4)	0.02

^a^Kruskal-Wallis test

### Tutor present in the room versus tutor present online

In a pairwise comparison between the two synchronous groups, the tutorial groups with the tutor present online (B) scored higher on cognitive aspects compared to the tutorial groups with the tutor present in the room (A) (Table 3).

**Table 3:** Comparison of synchronous groups’ TGEI scores

	(A) Tutor present in the room	(B) Tutor present online	***p***-value^a^
	median (Q1-Q3), *n* = 136	median (Q1-Q3) *n* = 17	
Cognitive aspects	23 (21–24)	24 (23.5–25)	0.01
Motivational aspects	27 (24–30)	28 (25.5–29.5)	n.s.
Demotivational aspects	10 (7–14)	8 (6–10.5)	n.s.
Overall rating of group productivity	4 (4–5)	4 (4–5)	n.s.

^a^Mann-Whitney U

### Comparison of synchronous and asynchronous groups

When merging the data from the two synchronous groups that had a tutor either present in the room (A) or online (B) and comparing to the asynchronous group with a consultant tutor (C), the synchronous group had significantly higher scores on cognitive aspects, motivational aspects and overall rating of group effectiveness (Table 4).

**Table 4:** Pairwise comparison of synchronous versus asynchronous groups on TGEI scores

	Synchronous groups (A) and (B) Median (Q1-Q3), *n* = 153	Asynchronous groups (C) Median (Q1-Q3), *n* = 68	***p***-value^a^
Cognitive aspects	23 (21–25)	22 (20–23.8)	0.002
Motivational aspects	27 (24–30)	25 (22–27)	0.001
Demotivational aspects	10 (7–13)	9 (7–13)	n.s.
Overall rating of group productivity	4 (4–5)	4 (3–4)	0.001

^a^Mann-Whitney U

### Psychometric evaluation

Reliability, as measured by Cronbach’s alpha, was adequate on both the subscale and the aggregated levels. All three subscales displayed adequate homogeneity (coefficient *H* > 0.3) when analysing each subscale separately. However, when taken together as a total summed score, the TGEI did not represent a homogeneous construct on this aggregated level (Table 5).

**Table 5:** Psychometric evaluation of the TGEI

Subscales	Homogeneity (Coef. H)	Cronbach’s alpha	Number of items	Number of cases^a^
Cognitive aspects	0.41	0.80	7	218
Motivational aspects	0.38	0.79	7	211
Demotivational aspects	0.40	0.73	5	221
Total TGEI^b^,	0.17	0.78	20	197

^a^Incomplete cases discarded for each scale

^b^Including the single ov;erall productivity rating item. Negative worded items 15–19 reversed

## Discussion

This study explored the effectiveness of tutorial groups with varied settings and tutor presence. The main results indicate that the tutor’s synchronous contribution is influential, regardless of the tutor’s online or face-to-face presence. The students’ prior PBL experience was not related to perceived effectiveness and neither were the motivational or cognitive aspects, as measured by the TGEI.

A tutor in the room did not seem to improve the students’ motivational and demotivational aspects, and it had only a minor influence on the cognitive aspects compared with online synchronous tutoring. This is in accordance with the results of De Jong et al. [18], who found face-to-face tutoring to be comparable with synchronous online tutorial sessions. However, the tutor’s synchronous presence, whether in the room or online, appears to be an influential aspect on the learning process. The students, however, reported less overall effectiveness and slightly lower motivation with a tutor as a consultant, i.e. when the tutor gave the students comments asynchronously on how they handled the scenarios and solved the clinical problems. In line with Slavin’s viewpoints on conceptual cooperative learning strategies [10], it could be argued that synchronous tutoring supports the students’ internal motivation and encourages participation and active involvement in the learning activities in a way that is impossible to achieve asynchronously.

Similar scores between the motivational and cognitive aspects in the groups were not surprising because the motivational and cognitive aspects are complementary and important for the learning outcomes [10]. The motivational aspects include the group positively influencing individual student learning and a feeling of a responsibility for the group to succeed, all factors that could differentiate between tutor in the room or tutor online PBL groups. The results of our study support the priority of synchronous PBL tutoring and suggest that campus-based face-to-face settings need not be crucial for the effectiveness of the group. Core processes in the PBL approach, such as active and self-directed learning and tutor roles, can just as effectively be supported online [24].

In our study, no differences were identified between students with or without prior PBL experience. One explanation might be that in all groups there was at least one student familiar with PBL, who could share experiences and guide the process when necessary. This collaborative effect is also relevant to other inequalities and is an inherent cornerstone of the PBL approach [8, 9]. Another explanation might be that the students were experienced as nurses and were therefore used to solving clinical problems as a team, which could have influenced their capacity to handle the PBL procedure that focuses on patient-centred scenarios.

The instrument for assessing PBL group effectiveness, the TGEI, is useful to evaluate important aspects of tutorial group work also in a Swedish setting. It was able to detect cognitive and motivational differences between the participant groups. The results show that the TGEI functioned well when analysing the three subscales separately, with acceptable reliability and homogeneity. However, despite good overall reliability, the sum scores on aggregated levels displayed low homogeneity. This indicates variations in what the subscales measure. Further research should investigate these differences before drawing far-reaching conclusions based on total sum scores. The expert group’s assessment of the translation, in addition to the student interviews, validated the item wording to an acceptable level. However, a more extensive evaluation of the wording with e.g. bachelor’s level student groups could further improve content validity.

The study has some limitations. The cross-sectional design was conducted in a regular educational setting, i.e. we did not assign students to the various settings in a controlled fashion, thereby reducing the possibility to draw conclusions about causal relationships. Moreover, the sample size differed between the groups, notably the online group B was comparatively small, which have to be considered when interpreting the findings. The participants were all studying at an advanced nursing level. These students are often well motivated, and our results might not be generalisable to students at other levels, such as bachelor’s level, or to longer study programmes with more extensive PBL tutorial work. The results might also not be generalisable to longer programmes, which provide more experiences of working in tutorial groups. As no similar instrument was found available in Swedish, no validity comparison were performed with similar instrument. The statistical analyses and psychometric evaluation was performed with a modest sample size (*n* = 210 for the scale analysis). For scale analysis using the Mokken approach (coefficient *H*) Watson et al. state that a minimum sample size of *n* = 250 is required [25], implying that our results should be interpreted with some caution.

## Conclusions

The tutor’s presence is productive for PBL group effectiveness with respect to cognitive and motivational aspects. However, the presence need not be in the actual room but can occur in online settings as long as the tutoring is not delayed in time. The TGEI is able to detect differences in perceived effectiveness in tutorial groups in a Swedish nursing education context. However, the total sum scores should be interpreted with caution because of low homogeneity between subscales.

## Data Availability

The datasets generated and/or analysed during the current study are not publicly available due to consent is not stating this, but are available from the corresponding author on reasonable request.

## Abbreviations

PBL: Problem-based learning
TGEI: The Tutorial Group Effectiveness Instrument
SD: Standard deviations

## References

[1] Savin-Baden M, Major CH (2004). Foundations of problem-based learning: McGraw-hill education (UK).

[2] Neville AJ (1999). The problem-based learning tutor: teacher? Facilitator? Evaluator?. Med Teach.

[3] Eckler U, Greisberger A, Höhne F, Putz P (2017). Blended learning versus traditional teaching-learning-setting: evaluation of cognitive and affective learning outcomes for the inter-professional field of occupational medicine and prevention/blended learning versus traditionelles Lehr-Lernsetting: Evaluierung von kognitiven und affektiven Lernergebnissen für das interprofessionelle Arbeitsfeld Arbeitsmedizin und Prävention. Int J Health Prof.

[4] Segerman J, Crable E, Brodzinski J (2016). E-learning and medical residents, a qualitative perspective. Inf Syst Educ J.

[5] Tudor Car L, Kyaw BM, Dunleavy G, Smart NA, Semwal M, Rotgans JI, Low-Beer N, Campbell J (2019). Digital problem-based learning in health professions: systematic review and meta-analysis by the digital health education collaboration. J Med Internet Res.

[6] Back DA, Haberstroh N, Antolic A, Sostmann K, Schmidmaier G, Hoff E (2014). Blended learning approach improves teaching in a problem-based learning environment in orthopedics-a pilot study. BMC Med Educ.

[7] Munro V, Morello A, Oster C, Redmond C, Vnuk A, Lennon S, Lawn S (2018). E-learning for self-management support: introducing blended learning for graduate students–a cohort study. BMC Med Educ.

[8] Taylor D, Miflin B (2008). Problem-based learning: where are we now?. Med Teach.

[9] Wood DF (2003). Problem based learning. BMJ (Clinical Res Ed).

[10] Slavin RE (1996). Research on cooperative learning and achievement: what we know, what we need to know. Contemp Educ Psychol.

[11] McConnell D (2005). Examining the dynamics of networked e-learning groups and communities. Stud High Educ.

[12] Ding Y, Zhang P (2018). Practice and effectiveness of web-based problem-based learning approach in a large class-size system: a comparative study. Nurse Educ Pract.

[13] Page J, Meehan-Andrews T, Weerakkody N, Hughes DL, Rathner JA (2017). Student perceptions and learning outcomes of blended learning in a massive first-year core physiology for allied health subjects. Adv Physiol Educ.

[14] Price Kerfoot B, Masser BA, Hafler JP (2005). Influence of new educational technology on problem-based learning at Harvard Medical School. Med Educ.

[15] Ellaway RH, Poulton T, Jivram T (2015). Decision PBL: a 4-year retrospective case study of the use of virtual patients in problem-based learning. Med Teach.

[16] Azer SA (2011). Introducing a problem-based learning program: 12 tips for success. Med Teach.

[17] Jin J, Bridges SM (2014). Educational technologies in problem-based learning in health sciences education: a systematic review. J Med Internet Res.

[18] De Jong N, Krumeich J, Verstegen DM (2017). To what extent can PBL principles be applied in blended learning: lessons learned from health master programs. Med Teach.

[19] Singaram VS, Van Der Vleuten CP, Van Berkel H, Dolmans DH (2010). Reliability and validity of a tutorial group effectiveness instrument. Med Teach.

[20] Shankar PR, Nandy A, Balasubramanium R, Chakravarty S (2014). Small group effectiveness in a Caribbean medical school’s problem-based learning sessions. J Rduc Eval Health Prof.

[21] Harkness J, Harkness J, Vijver FJR, Mohler PP (2002). Questionnaire translation. Cross-cultural survey methods.

[22] Stochl J, Jones PB, Croudace TJ (2012). Mokken scale analysis of mental health and well-being questionnaire item responses: a non-parametric IRT method in empirical research for applied health researchers. BMC Med Res Methodol.

[23] Streiner DL (2003). Starting at the beginning: an introduction to coefficient alpha and internal consistency. J Pers Assess.

[24] Boelens R, De Wever B, Rosseel Y, Verstraete AG, Derese A (2015). What are the most important tasks of tutors during the tutorials in hybrid problem-based learning curricula?. BMC Med Educ.

[25] Watson R, Egberink IJ, Kirke L, Tendeiro JN, Doyle F (2018). What are the minimal sample size requirements for Mokken scaling? An empirical example with the Warwick-Edinburgh mental well-being scale. Health Psychol Behav Med.

[26] CODEX: Ethical Review of Research. Rules and guidelines for research. The Swedish research council. http://www.codex.vr.se/en/manniska5.shtml. Accessed 10 Dec 2019.

